# Viable and Heat-Inactivated *Bifidobacterium bifidum* MIMBb75 Protect the Intestinal Barrier

**DOI:** 10.3390/microorganisms14061349

**Published:** 2026-06-16

**Authors:** Martin Storr, Martin Gschwender

**Affiliations:** 1 Zentrum für Endoskopie, MVZ Internistenzentrum Gauting-Starnberg, 82319 Starnberg, Germany; 2 Ärztliche Privatpraxis, 80469 München, Germany

**Keywords:** *B. bifidum* MIMBb75, probiotic, heat-inactivated, cell surface hydrophobicity, intestinal barrier, irritable bowel syndrome

## Abstract

Irritable bowel syndrome (IBS) is a common disorder of gut–brain interaction (DGBI) of multifactorial genesis. Studies consistently show a disrupted intestinal barrier with increased permeability in IBS patients, regardless of subtype. This allows facultative pathogenic bacteria to translocate into underlying body tissue and to initiate or exacerbate IBS symptoms. Protecting the intestinal barrier is therefore a primary therapeutic target. *Bifidobacterium bifidum* MIMBb75 has proven its efficacy in IBS both in its viable and heat-inactivated forms. Its efficacy is thought to be mediated by the physical adhesion of *B. bifidum* MIMBb75 to intestinal epithelial cells, thereby protecting the intestinal barrier. In the present study, we show—using a Caco-2 model—that this strain-specific adhesion is facilitated by the high cell surface hydrophobicity of *B. bifidum* MIMBb75, which is retained following heat inactivation. In line with these adhesive properties, both viable and heat-inactivated *B. bifidum* MIMBb75 protect the epithelial barrier, as indicated by an increased transepithelial electrical resistance in Caco-2 monolayers. Together, these findings strongly support a physical mode of action in which both viable and heat-inactivated *B. bifidum* MIMBb75 adhere to the epithelial surface and act, figuratively, as a protective plaster on the epithelial barrier.

## 1. Introduction

Irritable bowel syndrome (IBS) is a common disorder of gut–brain interaction (DGBI) affecting about 10% of the population worldwide [1,2]. Although the disease is of multifactorial genesis, biopsy examinations and lactulose-mannitol tests in IBS patients have consistently shown a damaged intestinal barrier at the epithelial cell layer with an increased intestinal permeability, independent of IBS subtype [3,4,5,6,7,8]. Moreover, it could be demonstrated that the severity of typical IBS symptoms, such as abdominal pain and diarrhea, is increased with increased intestinal permeability [6,9].

In a healthy intestine, a two-tiered mucus layer forms a physical barrier between the intestinal lumen and the epithelium, preventing contact of bacteria, antigens, toxins, and other extrinsic substances with the epithelial cells [10,11]. The inner mucus layer is entirely devoid of bacteria [12]. In IBS patients, however, this mucus layer is more permeable, exposing epithelial cells directly to bacteria, bacterial antigens, and other pathogenic substances [13].

Direct exposure of intestinal epithelial cells to pathogenic substances, such as lipopolysaccharides (LPS), initiates a pathogenic process and causes the release of pro-inflammatory mediators and reactive oxygen species (“oxidative stress”), as well as low-grade immune activation [14,15]. As a result, the structural integrity of the epithelial cells deteriorates further, and the intercellular tight junctions disintegrate, thereby providing a conduit for more facultative pathogenic bacteria to enter the host tissues [16,17,18,19]. Additionally, due to these host defense activities, the mucus layer is thinned and degraded [20], further perpetuating the disease process. One primary therapeutic target in IBS is therefore to protect the epithelial layer.

Recently, there has been increased interest in probiotic bacteria as a treatment of IBS that is generally regarded as safe—many carry the Qualified Presumption of Safety status assigned by the EFSA. However, only selected probiotic strains were proven to be effective [21,22,23,24,25,26], and even fewer are believed to protect the intestinal barrier [27,28]. In this context, *Bifidobacterium bifidum* MIMBb75, a strain originally isolated from human feces [29], is among the best-studied bacterial strains. *B. bifidum* MIMBb75 has proven its effectiveness in alleviating IBS and all its main symptoms, both in its viable [30] and heat-inactivated forms [31], and has recently been named in the German S3 IBS Clinical Practice Guideline [32]. *B. bifidum* MIMBb75 was suggested to have a strong adhesive property that exerts a protective effect on the intestinal barrier—yet its strain-specific mode of action in either form (viable/heat-inactivated) has not yet been comprehensively described in the literature.

In previous publications, viable *B. bifidum* MIMBb75 was shown to adhere to Caco-2 cells, a well-known and widely accepted model of the intestinal barrier, up to 500 times stronger compared to other known probiotic bacteria, including *B. animalis* subsp. *lactis* BB-12 and *L. rhamnosus* GG. Further, Andresen and co-workers noted that the adhesiveness of *B. bifidum* MIMBb75 is retained following heat inactivation [31]. The aim of this study is to investigate the protective effect of viable and heat-inactivated *B. bifidum* MIMBb75 on the epithelial barrier using a Caco-2 cell model.

## 2. Materials and Methods

### 2.1. Culturing of Caco-2 Cells

We validated the adhesion ability of heat-inactivated *B. bifidum* MIMBb75 on a Caco-2 cell line, a widely applied standard in vitro model of the human intestinal epithelium [33]. Caco-2 cells form fully differentiated, polarized epithelial monolayers, including apical brush border microvilli and tight junctions. Caco-2 cells (human colon adenocarcinoma cell line, CLS GmbH, Eppelheim Germany) were cultured in Minimum Essential Medium Eagle (CLS GmbH, Eppelheim Germany) or Dulbecco’s Modified Eagle Medium/Nutrient Mixture F-12 (PAN Biotech, Aidenbach Germany) supplemented with 10% (*v*/*v*) heat-inactivated fetal bovine serum (Gibco, Thermo Fisher Scientific, Waltham, MA, USA) and 1% (*v*/*v*) antibiotics solution (penicillin 10,000 U/mL and streptomycin 10 mg/mL, Gibco, Thermo Fisher Scientific, Waltham, MA, USA) at 37 °C in a humidified atmosphere (95% humidity) with 5% CO_2_. The culture medium was replaced every 2–3 days. At 70–80% confluence, cells were passaged (every 2–3 days) by trypsinization using a 0.05% trypsin-EDTA solution (Gibco, Thermo Fisher Scientific, Waltham, MA, USA) for 10 min and re-diluted in a medium well plate for seeding.

Clinical evidence suggests that the intestinal mucus barrier is often significantly thinned or compromised in the context of IBS [34]. To focus specifically on the direct interaction between *B. bifidum* MIMBb75 and the epithelial cell surface, we deliberately chose a Caco-2 monoculture rather than a mucin-producing Caco-2/HT29-MTX co-culture.

### 2.2. Culturing of Bacteria

*B. bifidum* MIMBb75 or *B. animalis* subsp. *lactis* BB-12 were grown on separate MRS agar plates (Thermo Scientific, Waltham, MA, USA) for at least 24 h at 37 ± 2 °C under an anaerobic atmosphere. For experiments, 50 mL of MRS broth (Dr. Möller & Schmelz, Göttingen, Germany) supplemented with 0.5 g/L L-cysteine hydrochloride (Sigma Aldrich, Taufkirchen, Germany) was inoculated from the plates and incubated for a max of 24 h at 37 ± 2 °C under anaerobic conditions. The bacteria were centrifuged at 7000 rpm for 10 min and washed once with sterile phosphate-buffered saline (PBS, pH 7.2) prior to resuspending in PBS. The number of bacterial cells was determined by spectroscopy using optical density at a 600 nm wavelength (OD_600_).

*L. rhamnosus* GG was grown on MRS agar plates for 24 h at 37 ± 2 °C in an anaerobic atmosphere. *E. coli* Nissle 1917 was grown on CASO agar plates (bioMérieux, Marcy-l’Étoile, France) for 24 h at 30–35 °C under aerobic conditions. For experiments, the bacteria were flushed off the agar plates using sterile PBS. The bacterial suspension was collected, centrifuged at 7000 rpm for 10 min, and washed once with PBS. The number of bacteria was determined by measuring OD_600_.

### 2.3. Heat Inactivation of B. bifidum MIMBb75

To obtain heat-inactivated *B. bifidum* MIMBb75, viable cells of *B. bifidum* MIMBb75 were subjected to a proprietary time- and temperature-controlled heat inactivation process that preserves the morphology of the bacteria, as confirmed by light microscopy. Successful heat inactivation was confirmed by >99.9% growth inhibition on MRS agar plates across several dilutions of the heat-treated bacterial suspension. The bacterial concentration used for the inactivated preparation was calculated based on the viable cell count prior to inactivation and is reported as cells/well rather than CFU/well.

### 2.4. Adhesion Assay and Light Microscopy

Caco-2 cells were seeded at a density of 3 × 10^5^ cells/well on untreated tissue culture 24-well Transwell inserts (0.47 cm^2^) growth area (Corning, Corning, NY, USA) and incubated at 37 °C in an atmosphere of 95% humidity and 5% CO_2_ for 21 days.

The cell monolayers were carefully washed twice with PBS (pH 7.4) before adding approximately 1 × 10^7^ CFU/well of viable *B. bifidum* MIMBb75 or 1 × 10^7^ cells/well of heat-inactivated *B. bifidum* MIMBb75, respectively, resuspended in antibiotic-free medium. After 1 h of incubation at 37 °C in an atmosphere of 95% humidity and 5% CO_2_, all monolayers were washed three times with PBS to release unbound bacterial cells. The monolayers were fixed with 2 mL of methanol for 8 min at room temperature. After methanol was removed, cells were stained with 3 mL of 1:20 Giemsa stain solution (Carlo Erba, Cornaredo, Italy) and incubated in the dark for 30 min at room temperature. Wells were then washed at least three times with PBS until no color was observed in the washing solution and dried in an incubator at 30 °C for 1 h. Microscope cover glasses were removed and examined using phase-contrast light microscopy (magnification of 40×; Di-Li 2025, Distelkamp-Electronic, Kaiserslautern, Germany). The adhesion indices of viable and heat-inactivated *B. bifidum* MIMBb75 were determined by counting attached bacterial cells to 100 Caco-2 cells. Counting was performed by Tentamus (Berlin, Germany) as an external CRO by a trained microbiologist in 20 randomly selected microscope fields per cover glass at 40× magnification. The applied bacterial dose corresponds to 2.13 × 10^5^ cells/mm^2^ of Caco-2 monolayer (1 × 10^7^ cells per 0.47 cm^2^ insert). Cell numbers of heat-inactivated *B. bifidum* MIMBb75 were calculated from OD_600_ measurements using an internally calibrated factor of OD_600_ 1.0 = 1 × 10^8^ cells/mL, derived from serial plating and colony counting of the corresponding viable preparation prior to inactivation.

### 2.5. Determination of Cell Surface Hydrophobicity

Cell surface hydrophobicity (CSH) is a strong predictor of bacterial adhesion to Caco-2 cells [35]. We evaluated the CSH of viable and heat-inactivated *B. bifidum* MIMBb75 and of other probiotic strains (*L. rhamnosus* GG, *B. animalis* subsp. *lactis* BB-12 and *E. coli* Nissle 1917) using the *n*-hexadecane extraction method [35]. To this end, we measured the absorbance at 560 nm (A_560nm_) of the aqueous phase before and after extraction with *n*-hexadecane. In detail, the bacteria suspension was centrifuged at 7500 rpm for 7 min, washed three times with PBS (pH 7.4), and resuspended in the same buffer. The bacteria suspension was then diluted and, by measurement of A_560nm_ using the Fluidlab R-300 device (Anvajo, Dresden, Germany), adjusted to OD»0.6. Subsequently, 2 mL of the bacterial suspension was mixed with the corresponding volume of *n*-hexadecane (Sigma-Aldrich, Taufkirchen, Germany) in a 5 mL Eppendorf tube (Eppendorf, Hamburg, Germany) and mixed vigorously for 2 min using a vortexer (Phoenix Instrument, Garbsen, Germany). The following ratios of *n*-hexadecane/bacteria suspension were measured: 0.1; 0.2; 0.5; 1.0; 2.0. Following a settling time of 2 min at room temperature, 1 mL of the lower, aqueous phase was carefully transferred to a cuvette, and A_560nm_ was determined again.

The decrease in the absorbance value was taken as a measure for CSH and was calculated as follows:

CSH = [(A_0_ − A)/A_0_] × 100 (1)

where A_0_ and A were absorbance values before and after extraction of the bacteria with *n*-hexadecane. Cell-free PBS buffer served as the spectrometer blank to correct for background absorbance and yielded an OD of 0.00115 ± 0.00383.

### 2.6. Caco-2 Transwell Cultures and Measurement of Transepithelial Electrical Resistance

To assess the protective properties of viable and heat-inactivated *B. bifidum* MIMBb75 on an intact and damaged intestinal barrier, we measured Caco-2 cell layer permeability using transepithelial electrical resistance (TEER). TEER, a widely applied measurement to investigate intestinal barrier function in vitro, is the electrical resistance measured across a cellular monolayer in response to an applied current that depends on the resistances of the transcellular and paracellular pathways [36]. The degree of epithelial permeability can be expressed by the ratio of the two resistances. The lower the paracellular resistance compared to transcellular resistance, the more permeable the epithelial monolayer [37].

Two separate experiments were conducted with transwell cultures: one Caco-2 cell culture without disruptors and one with disruptors [*E. coli* LPS/hydrogen peroxide (HPO)]. The effects of *E. coli* LPS and HPO on Caco-2 cell membrane permeability have previously been explored in various in vitro studies [38,39,40,41]. *E. coli* LPS in the apical compartment imitates mechanisms of microbial dysbiosis and disrupts epithelial barrier proteins as a result of the subsequent inflammatory response [39,41], whereas HPO in the basolateral medium was used as a model of sub-mucosal inflammation in IBS and induces, amongst others, oxidative stress, ultimately disrupting the epithelial barrier proteins [38,40].

Caco-2 cells were seeded on polyester (PET) membrane Transwell inserts (24-well format, 0.47 cm^2^ growth area, 0.4 µm pore size; Corning, Corning, NY, USA) at a density of 7 × 10^4^ cells/well (1.5 × 10^5^ cells/cm^2^). The same insert format was used for both experimental arms (without disruptors and with disruptors [*E. coli* LPS/hydrogen peroxide]). In each plate, one well was left cell-free and served as a blank for TEER measurements. The cells were cultured at 37 °C in an atmosphere of 95% humidity and 5% CO_2_ for 21 days until fully differentiated. The culture medium was changed every 2–3 days. TEER was measured using an ohm voltmeter equipped with a chopstick electrode (EVOM^2^, World Precision Instruments, Friedberg, Germany, or Millicell-ERS-2, Merck Millipore, Burlington, MA, USA) to monitor its development during cell differentiation. Only morphologically and physiologically well-developed Caco-2 cell monolayers with TEER values around 1200 Ω were used for the experiments. On day 21, before any experiment, the medium was replaced with fresh, antibiotic-free medium in both apical and basolateral chambers.

In the experiment without disruptors, a baseline TEER was measured, and the apical medium was replaced with fresh medium containing either 1 × 10^7^ CFU/well of viable *B. bifidum* MIMBb75, or 1 × 10^7^ cells/well of heat-inactivated *B. bifidum* MIMBb75, or medium without bacteria (untreated control). This concentration was selected as an experimentally established dose commonly applied in comparable in vitro adhesion and epithelial barrier assays [42,43,44,45,46]. Subsequent TEER measurements were performed for t = 1, 2, 3, 4, 5, 6, and 24 h.

In the LPS experiment, an initial TEER was measured, and the apical medium was replaced with 500 µL of medium containing 24 ng/mL LPS (*E. coli* 0113, K-endotoxin, BRP batch 5). The cells were incubated for approx. 3 h at 37 °C (95% humidity, 5% CO_2_). The apical medium was then exchanged with medium containing either 1 × 10^7^ CFU/well of viable *B. bifidum* MIMBb75, or 1 × 10^7^ cells/well of heat-inactivated *B. bifidum* MIMBb75, or medium without bacteria (LPS-only control). The baseline (t = 0 h) TEER was measured immediately after the exchange of medium, and subsequent TEER measurements were performed for t = 1, 2, 3, 4, 5, 6, and 24 h.

In the HPO experiment, an initial TEER was measured before the basolateral medium was replaced with 1 mL of medium containing 50 mM HPO (Carl Roth, Karlsruhe, Germany). Cells were incubated for approx. 30 min at 37 °C (95% humidity, 5% CO_2_). After the incubation period, the basolateral medium was replaced with fresh medium. The apical medium was then exchanged with medium containing either 1 × 10^7^ CFU/well of viable *B. bifidum* MIMBb75, or 1 × 10^7^ cells/well of heat-inactivated *B. bifidum* MIMBb75, or no bacteria (HPO–only control). Again, baseline (t = 0 h) TEER was measured immediately after the exchange of the medium, and subsequent TEER measurements were performed for t = 1, 2, 3, 4, 5, 6, and 24 h.

Net TEER values (Ω × cm^2^) were obtained by subtracting the TEER of the blank and multiplying it by the surface area of the wells. To determine the change in TEER over time, the obtained net TEER values were normalized to baseline.

### 2.7. Statistical Analysis

The data are given in terms of mean ± standard deviation (SD). For the determination of the hydrophobicity, an ANOVA with all pairwise comparisons and Holm-Bonferroni Type I corrections was applied, testing the differences between the average *n*-hexadecane/bacteria suspension ratio of each strain. Statistical differences in the TEER experiments were evaluated using a two-tailed unpaired t-test (predefined function in Microsoft Excel, Microsoft Corporation, Redmond, WA, USA). A *p*-value < 0.05 was considered statistically significant.

## 3. Results

### 3.1. Both Viable and Heat-Inactivated B. bifidum MIMBb75 Show Strong Adherence to Caco-2 Cells Through Hydrophobic Interactions

**Figure 1:**
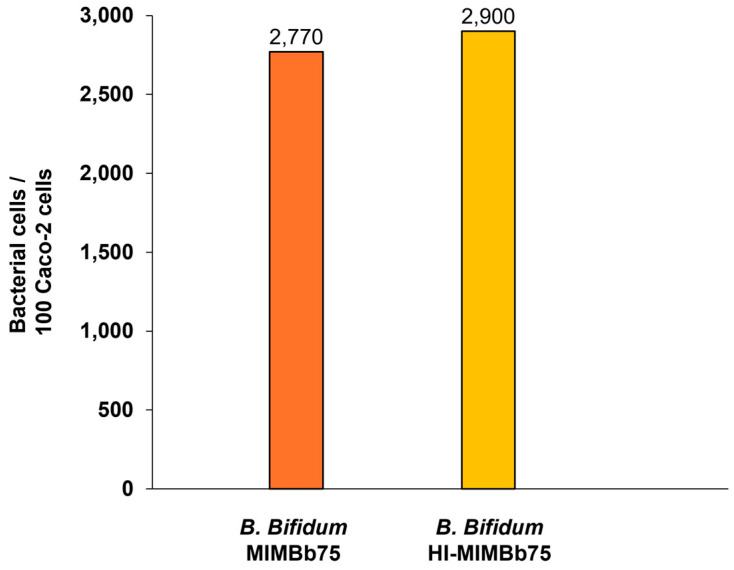
Adhesion index of viable and heat-inactivated *B. bifidum* MIMBb75 adhering to 100 Caco-2 cells.

We observed that viable and heat-inactivated *B. bifidum* MIMBb75 possess a similarly high physical adhesion ability: The adhesion index, i.e., the number of bacterial cells firmly adhering after at least three washing steps to a set of 100 Caco-2 cells, was 2770 for viable *B. bifidum* MIMBb75 and 2900 for heat-inactivated *B. bifidum* MIMBb75 (Figure 1).

**Figure 2:**
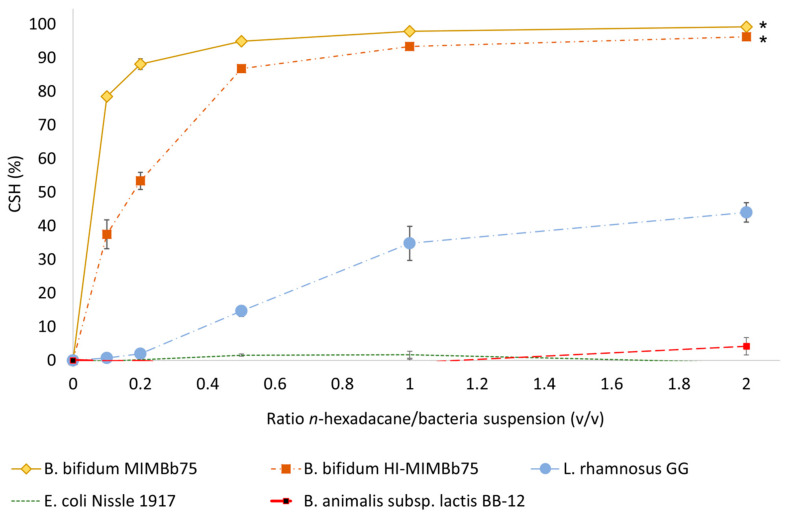
Cell Surface Hydrophobicity (CSH) of viable *B. bifidum* MIMBb75 and heat-inactivated *B. bifidum* MIMBb75 compared to *L. rhamnosus* GG, *B. animalis* subsp. *lactis* BB-12 and *E. coli* Nissle 1917, respectively, as assessed by relative absorbance rates at 560 nm of increasing *n*-hexadecane/bacteria suspension extraction ratios. Results are shown as mean ± SD (n = 3). * CSH values of viable and heat-inactivated *B. bifidum* MIMBb75 do not differ significantly from each other (*p* = 0.2492), but they do pairwise from all others (*p* < 0.0001).

Viable and heat-inactivated *B. bifidum* MIMBb75 revealed by far the highest CSH when compared to *L. rhamnosus* GG, *B. animalis* subsp. *lactis* BB-12 and *E. coli* Nissle 1917, all of which have been successfully used in the treatment of IBS [47,48,49,50,51] (Figure 2).

*L. rhamnosus* GG, *B. animalis* subsp. *lactis* BB-12 and *E. coli* Nissle 1917 were used as well-characterized benchmark strains to contextualize the CSH of *B. bifidum* MIMBb75, not as definitive positive or negative controls. Because CSH is a continuous physical property, values can vary substantially even among closely related strains. Accordingly, Pan et al. reported CSH values ranging from 0.30% to 37.24% across 23 bifidobacterial strains [35]. For both viable and heat-inactivated *B. bifidum* MIMBb75, the aqueous-phase absorbance decreased progressively with increasing *n*-hexadecane/bacterial suspension ratios, approaching maximal extraction at the highest ratios.

Already at very low extraction ratios of 0.1 and 0.2, both viable and heat-inactivated *B. bifidum* MIMBb75 show a hydrophobic affinity to *n*-hexadecane that is several-fold higher than for any of the other tested bacteria (including another bifidobacterium strain, *B. animalis* subsp. *lactis* BB-12). The only other strain showing a moderate CSH is *L. rhamnosus* GG (fluid-fluid interface absorbance of 44% at an extraction ratio of 2.0). In contrast, *E. coli* Nissle 1917 and *B. animalis* subsp. *lactis* BB-12 do not appear to be hydrophobic.

### 3.2. Both Viable and Heat-Inactivated B. bifidum MIMBb75 Increase TEER

**Figure 3:**
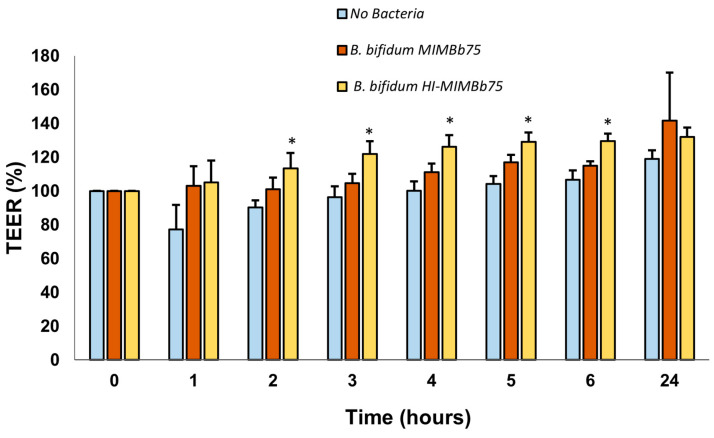
Change in intestinal barrier function by viable *B. bifidum* MIMBb75 (10^7^ CFU/well) and heat-inactivated *B. bifidum* MIMBb75 (10^7^ cells/well), in comparison to the untreated cell control (medium without bacteria), as indicated by an increase in TEER over 24 h measured in Caco-2 transwell bacterial co-cultures. Results are normalized to baseline and shown as mean ± SD (n = 2–5). * *p* < 0.05 versus untreated control.

In the experiment without any disruptors, TEER (normalized to baseline) of Caco-2 cells exposed to viable or heat-inactivated *B. bifidum* MIMBb75 was consistently higher than the untreated control (medium without bacteria) in all measurements. At 24 h, the TEERs (normalized to baseline) of Caco-2 cells exposed to viable *B. bifidum* MIMBb75 increased to 141.78 ± 28.31% (*p* = 0.40 vs. untreated control 118.90 ± 5.11%), while the TEER of Caco-2 cells exposed to heat-inactivated *B. bifidum* MIMBb75 increased to 132.07 ± 5.38% (*p* = 0.05 vs. untreated control 118.90 ± 5.11%) (Figure 3).

**Figure 4:**
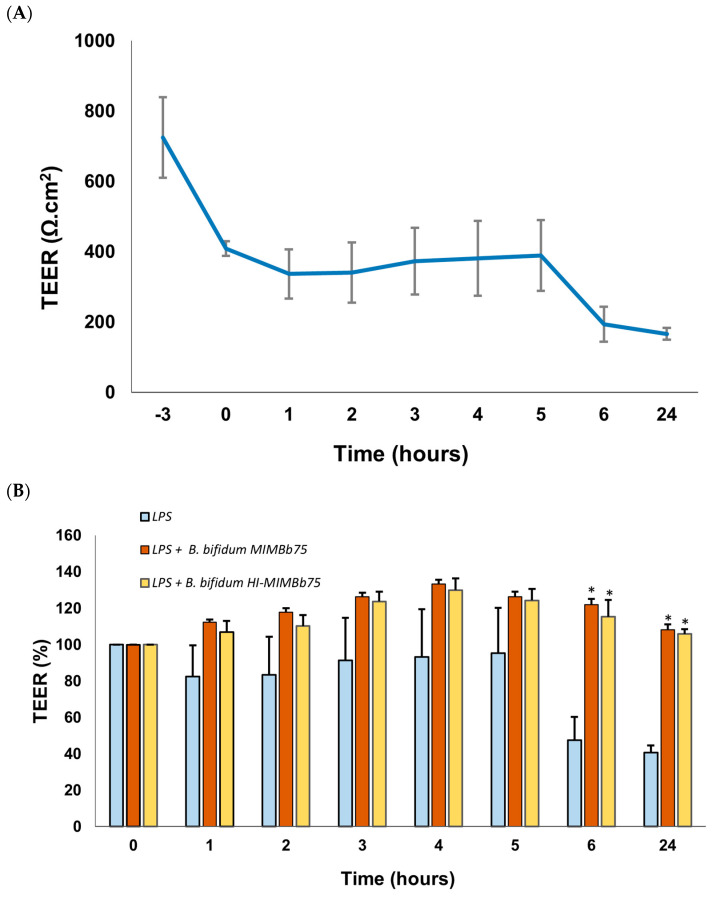
(**A**) Caco-2 cells were pre-treated with 24 ng/mL *E. coli* LPS and incubated for 3 h. During the incubation period, TEER dropped from 725.18 ± 114.76 Ω × cm^2^ to 408.40 ± 20.77 Ω × cm^2^. (**B**) Addition of 10^7^ CFU/well of viable *B. bifidum* MIMBb75 or 10^7^ cells/well of heat-inactivated *B. bifidum* MIMBb75 to LPS-pre-treated cells increased intestinal barrier function as measured by TEER (normalized to baseline), whereas it dropped to 40.71 ± 3.95% for the LPS-only control (medium without bacteria). Results are shown as mean ± SD (n = 3). * *p* < 0.05 versus LPS-only control.

Treating Caco-2 cells with 24 ng/mL LPS induced a TEER drop from 725.18 ± 114.76 Ω × cm^2^ to 408.40 ± 20.77 Ω × cm^2^ during the incubation period of three hours. Afterwards, TEER was almost stable at a value around 370 Ω × cm^2^ for the first five hours, before decreasing to 166.11 ± 16.68 Ω × cm^2^ in 24 h (Figure 4A). *E. coli* LPS significantly disrupted the membrane integrity of Caco-2 cells at a 24 ng/mL concentration.

Upon addition of viable or heat-inactivated *B. bifidum* MIMBb75 to the LPS pretreated cells at t = 0, TEER (normalized to baseline, t = 0) at 24 h (Figure 4B) was significantly higher than the LPS-only control (medium-without bacteria) for both viable *B. bifidum* MIMBb75 (108.22 ± 2.90% vs. 40.71 ± 3.95%, *p* < 0.001) and heat-inactivated *B. bifidum* MIMBb75 (105.96 ± 2.64% vs. 40.71 ± 3.95%, *p* < 0.001).

**Figure 5:**
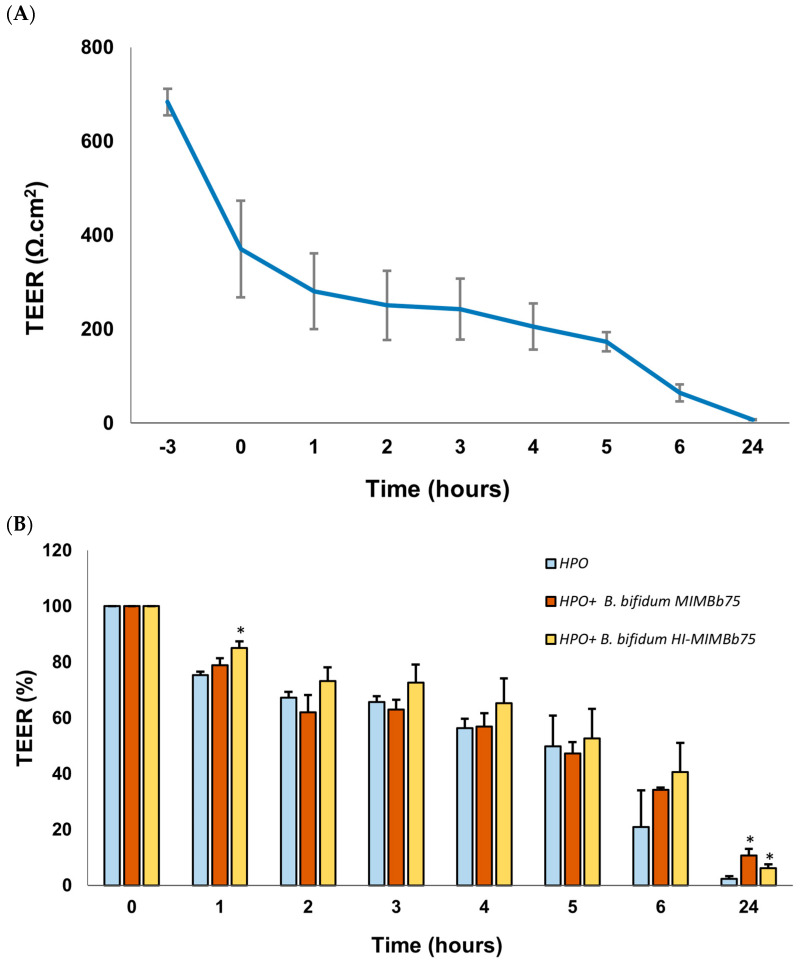
(**A**) Caco-2 cells were pre-treated with 50 mM HPO and incubated for 0.5 h. During the incubation period, TEER dropped from 683.43 ± 28.39 Ω × cm^2^ (measured initially 2.5 h before adding HPO) to 370.72 ± 102.73 Ω × cm^2^. (**B**) TEER (%) dropped to 2.31 ± 0.97% for the HPO-only control (medium without bacteria), whereas it dropped to 12.14 ± 1.45% and 6.2 ± 1.35% with the addition of 10^7^ CFU/well of viable *B. bifidum* MIMBb75 or 10^7^ cells/well of heat-inactivated *B. bifidum* MIMBb75. Results are shown as mean ± SD (n = 3). * *p* < 0.05 versus HPO-only control.

Unlike LPS, the addition of 50 mM HPO reduced TEER continuously throughout the entire measurement period from an initial 683.43 ± 28.39 Ω × cm^2^ to 7.57 ± 0.84 Ω × cm^2^ (Figure 5A). Over the course of 24 h, TEER (normalized to baseline, t = 0) in pre-treated Caco-2 cells decreased to 2.31 ± 0.97%. In comparison, TEER was roughly three to five times higher for cells treated with either viable or heat-inactivated *B. bifidum* MIMBb75 at the end of measurement (12.14 ± 1.45%; *p* = 0.006 vs. HPO-only control and 6.20 ± 1.35%; *p* = 0.030 vs. HPO-only control) (Figure 5B).

## 4. Discussion

IBS is a common gastrointestinal disease of multifactorial genesis. Various studies have consistently found a disrupted intestinal barrier with increased permeability in patients with IBS, irrespective of the underlying IBS subtype. Increased intestinal permeability contributes to the translocation of luminal bacterial antigens, which in turn causes irritation of the intestinal nervous system [9,16,18,19,52,53,54,55]. In consequence, the typical symptoms of IBS, such as abdominal pain and diarrhea, exacerbate. Protecting the intestinal barrier is hence a key target within IBS treatment. In the present study, we demonstrated that both viable and heat-inactivated *B. bifidum* MIMBb75 physically adhere to intestinal epithelial cells in high numbers and protect both the intact and the damaged epithelial barrier. To our knowledge, this is the first systematic study that systematically investigates the barrier-protecting effects of viable and heat-inactivated *B. bifidum* MIMBb75 within the same experimental framework.

Gentle heating leads to the inactivation of probiotic bacteria, preserving their overall structure but eliminating their ability to grow and multiply. Heat-inactivated probiotics present notable advantages with respect to standardization, transport, and storage stability [56]. For many years, cell viability has been regarded as one of the key—if not the single most crucial—properties of probiotics thought to underlie their health benefits [57,58,59,60,61,62]. However, evidence indicating that microbial viability is not essential for the efficacy has been demonstrated for several strains and conditions [63,64,65].

Both viable and heat-inactivated *B. bifidum* MIMBb75 show a distinctly high adherence to Caco-2 cells. Evaluation by light microscopy showed that the adhesion index, i.e., the number of bacterial cells adhering to an average set of 100 Caco-2 cells, was 2770 for viable *B. bifidum* MIMBb75 and 2900 for heat-inactivated *B. bifidum* MIMBb75, in line with previous findings on clinical efficacy by Guglielmetti [30] and Andresen [31]. The adhesion index of *B. bifidum* MIMBb75 is up to 500-fold higher compared to adhesion indices reported by others under comparable experimental conditions for other known probiotic bacteria, including *B. animalis* subsp. *lactis* BB-12 and *L. rhamnosus* GG [29]. These data confirm that physical adhesion to the intestinal epithelial tissue is a characteristic feature of *B. bifidum* MIMBb75 that is fully maintained upon heat inactivation.

Various mechanisms have been proposed by others to explain the forces driving this strong adhesion ability. In particular, an involvement of the specific surface protein BopA was initially hypothesized [29,66] but was subsequently disproven [67]. In contrast, our study indicates that the adhesion to the disrupted intestinal barrier is primarily mediated by the high CSH of both viable and heat-inactivated *B. bifidum* MIMBb75 through a physical mode of action.

Our results regarding the high CSH of viable and heat-inactivated *B. bifidum* MIMBb75 and its strong adhesion abilities to Caco-2 cells are consistent with a previous report of Pan and co-workers [35], who found a strong positive correlation between the two metrics. Indeed, already at very low extraction ratios of 0.1 and 0.2, viable and heat-inactivated *B. bifidum* MIMBb75 both show a hydrophobic affinity to *n*-hexadecane that is several-fold higher compared to other strains tested, including *B. animalis* subsp. *lactis* BB-12, which pertains to the same genus of bifidobacteria. These findings suggest that hydrophobic interactions—purely physical forces—are the primary mechanism underlying the adherence of viable and heat-inactivated *B. bifidum* MIMBb75 to epithelial cells.

Comparable to the findings by Briske-Anderson and co-workers [68], TEER values measured in this study were stable and only increased minimally in fully differentiated, untreated Caco-2 cells at day 21. However, following the addition of either viable or heat-inactivated *B. bifidum* MIMBb75, a marked increase in TEER values was observed. The consistently higher TEER values were also observed when the Caco-2 monolayers were pre-treated with LPS and HPO. The adhesion of both viable and heat-inactivated *B. bifidum* MIMBb75 to Caco-2 cells is in accordance with previous hypotheses on the clinical effect of *B. bifidum* MIMBb75 on the intestinal barrier in IBS patients [30,31].

One limitation of this study is that our findings are based on in vitro data using a Caco-2 cell model. However, Caco-2 monolayers are an established and widely accepted standard model for studying direct interactions between probiotic bacteria and the intestinal epithelium [69,70,71,72,73,74], and they are recognized by regulatory authorities as supportive mechanistic evidence. Conducting an invasive clinical trial for the sole purpose of elucidating the mode of action would, moreover, not be ethically justifiable, particularly given that the clinical efficacy of both viable and heat-inactivated *B. bifidum* MIMBb75 has already been demonstrated in two independent double-blind randomized clinical trials [30,31].

The use of a Caco-2 monolayer model is also consistent with the pathophysiological situation in IBS patients, in whom the protective mucus layer is significantly thinner than in healthy controls [34], so that direct bacteria–epithelium interactions are of particular physiological relevance.

Another limitation of the present study is that epithelial barrier integrity was assessed using a single functional readout. TEER is a well-established and widely accepted method to evaluate epithelial monolayer integrity in Caco-2 models and was therefore appropriate for the objective of this study. Nevertheless, the inclusion of complementary barrier assays would further strengthen the evidence for the observed barrier-stabilizing effect and should be considered in future studies.

A further limitation is the limited number of biological replicates in the TEER experiments, which reflects the strict inclusion criteria applied.

While the aim of the present study was to explicitly compare and examine the hydrophobicity and the protective effect of both viable and heat-inactivated *B. bifidum* MIMBb75, it might also be useful in the in vitro screening of other future probiotic treatments for IBS. Our results underpin the validity of the strong correlation between the CSH of bifidobacteria and their adhesiveness to the disrupted epithelium.

## 5. Conclusions

In summary, our results clearly demonstrate that *B. bifidum* MIMBb75—in both the viable and heat-inactivated forms—adheres exceptionally well to intestinal epithelial cells. Together with a significant increase in TEER values after the addition of viable and heat-inactivated *B. bifidum* MIMBb75, this suggests a protection of the intestinal epithelial barrier and provides mechanistic support for the efficacy of *B. bifidum* MIMBb75 in previously conducted clinical studies. Its adhesion is primarily mediated through physical forces for both viable and heat-inactivated *B. bifidum* MIMBb75, i.e., their extraordinarily high cell surface hydrophobicity, figuratively comparable to a physical plaster. Our findings also confirm that the barrier-protective capacity is independent of viability and, hence, metabolic activity. The strong adhesion of both viable and heat-inactivated *B. bifidum* MIMBb75 forms a protective layer above the epithelial surface and results in a significant increase in TEER, indicating protection of the intestinal epithelial barrier.

## Data Availability

The original contributions presented in this study are included in the article. Further inquiries can be directed to the corresponding author.

## Abbreviations

The following abbreviations are used in this manuscript:

A	Absorbance values after extraction of bacteria with *n*-hexadecane
A_0_	Absorbance values before extraction of bacteria with *n*-hexadecane
ANOVA	Analysis of variance
*B. animalis* subsp. *lactis* BB-12	*Bifidobacterium animalis* subspecies *lactis* BB-12
*B. bifidum*	*Bifidobacterium bifidum*
*B. bifidum* MIMBb75	Viable *Bifidobacterium bifidum* MIMBb75
*B. bifidum* HI-MIMBb75	Heat-inactivated *B. bifidum* MIMBb75
CFU	Colony-forming units
CSH	Cell surface hydrophobicity
DGBI	Disorder of gut–brain interaction
*E. coli*	*Escherichia coli*
HPO	Hydrogen peroxide
IBS	Irritable Bowel Syndrome
LPS	Lipopolysaccharide(s)
*L. rhamnosus* GG	*Lactobacillus rhamnosus* GG
OD	Optical density
SD	Standard deviation
TEER	Transepithelial electrical resistance
